# Reduced Effective Oxygen Delivery and Ventilation with a Surgical Facemask Placed under Compared to over an Oxygen Mask: A Comparative Study

**DOI:** 10.1155/2022/4798993

**Published:** 2022-01-21

**Authors:** Marvin G. Chang, Takashi Sakano, Benjamin S. Levin, David Convissar, Edward A. Bittner

**Affiliations:** Department of Anesthesia, Critical Care, and Pain Medicine, Massachusetts General Hospital, Boston, MA, USA

## Abstract

**Objectives:**

Consensus guidelines for perioperative anesthesia management during the COVID-19 pandemic recommend that patients wear a facemask in addition to their oxygen mask or nasal cannulae following tracheal extubation, where this is practical. The effects on effective oxygen delivery and ventilation of a surgical facemask under compared to over an oxygen (O_2_) mask are unclear.

**Design:**

Single-center, comparative pilot study. *Setting*. Endoscopy procedure room at a major academic hospital.

**Subjects:**

Five healthy anesthesiologists. *Interventions*. Using a carbon dioxide (CO_2_) sampling line positioned at the lips, the fraction of inspired O_2_ (FiO_2_), fraction of expiratory O_2_ (FeO_2_), expiratory end-tidal CO_2_ (EtCO_2_), and respiratory rate (RR) were measured under the following conditions: (1) a surgical facemask only, (2) a surgical facemask under an O_2_ mask, (3) an O_2_ mask only, and (4) a surgical facemask over an O_2_ mask. *Measurements and Main Results*. The sampled fractional expired oxygen (FeO_2_) at the lips was significantly lower when the surgical facemask was under compared to when over the O_2_ mask (27.9± 1.68 vs. 49.9 ± 6.27, *p* = 0.001), while there was no significant difference in inspired oxygen (FiO_2_). The sampled expiratory EtCO_2_ was significantly higher when the surgical facemask was under the O_2_ mask compared to when over the O_2_ mask (28.3 ± 8.5 vs. 23.5 ± 7.6, *p* = 0.026). The RR was not significantly different when the surgical facemask was under compared to over the O_2_ mask.

**Conclusions:**

Effective oxygen delivery and ventilation was reduced (lower FeO_2_ and increased EtCO_2_) when a surgical facemask was placed under compared to over an O_2_ mask.

## 1. Introduction

Surgical patients routinely require supplementary oxygen (O_2_) by facemask after undergoing anesthesia. Administration begins in the operating room, continues during transport to the recovery room, and extends for a period during recovery. Patients with COVID-19 undergoing surgery also commonly need supplementary O_2_ for a period of time following anesthesia yet pose a risk of viral spread. A simple O_2_ mask which is routinely used at the time of extubation and during the postoperative period has been shown to increase aerosolization of infectious viral particles that can be detected up to a distance of 0.4 meters at normal flow rates [1]. Given the infectious risk, consensus guidelines for anesthesia management during the COVID-19 pandemic recommend that patients wear a surgical facemask in addition to their O_2_ mask or nasal cannulae following tracheal extubation, where practical [2]. An unanswered question is whether the facemask should be placed over an O_2_ mask or if it should be worn underneath the O_2_ mask and if there is any difference in O_2_ delivery and carbon dioxide (CO_2_) elimination between the two configurations. A published letter assessing a single individual indicates that inspired O_2_ concentration may be equivalent with the two mask configurations; however, expired O_2_ concentration and carbon dioxide levels were not assessed [3]. We hypothesized that a surgical facemask worn underneath the O_2_ mask would both decrease the expired fraction of expiratory O_2_ concentration and carbon dioxide (CO_2_) levels. We sought to examine the various effects on oxygenation and ventilation of patients with the surgical facemask under or over the O_2_ mask and showed for the first time the consequences of reduced fraction of expiratory oxygen (FeO_2_) and increased expiratory end-tidal CO_2_ (EtCO_2_) with the surgical facemask under compared to over an O_2_ mask in health volunteers. Answering these questions could be important for surgical patients following extubation and during recovery from anesthesia, especially those with underlying respiratory impairment while we continue to minimize risk for aerosolization.

## 2. Methods

The study cohort consisted of five healthy anesthesiologists from our institution that volunteered to participate in the study. We performed a power calculation based on the Blinks et al.'s [3] study and used an expected mean and standard deviation of the differences in the EtCO_2_ of 15 and 6, respectively, and an alpha of 0.05 and a desired power of 0.8. The study received IRB exemption as a quality improvement project. Using a Drager Apollo Anesthesia machine in the endoscopy procedure room, we used a gas sampling line positioned at the lips to record the fraction of inspired oxygen (FiO_2_), fraction of expiratory oxygen (FeO_2_), expiratory end-tidal CO_2_ (EtCO_2_), and respiratory rate (RR) under the following conditions: (1) a surgical facemask only, (2) a surgical facemask under an O_2_ mask, (3) an O_2_ mask only, and (4) a surgical facemask over an O_2_ mask. O_2_ was delivered at 6 L/min via the oxygen mask. The subjects were under each condition for at least 2 minutes prior to making any measurements. For each condition, five individual measurements were recorded for FiO_2_, FeO_2_, EtCO_2_, and RR.

Descriptive statistics were performed and reported as a mean and standard deviation. Paired *t*-testswere used to perform comparisons between groups. A two-sided *p* value of <0.05 was used to denote statistical significance. All statistical analyses were performed using STATA® release 14.2 (StataCorp LLC, College Station, TX, USA).

## 3. Results

**Figure 1:**
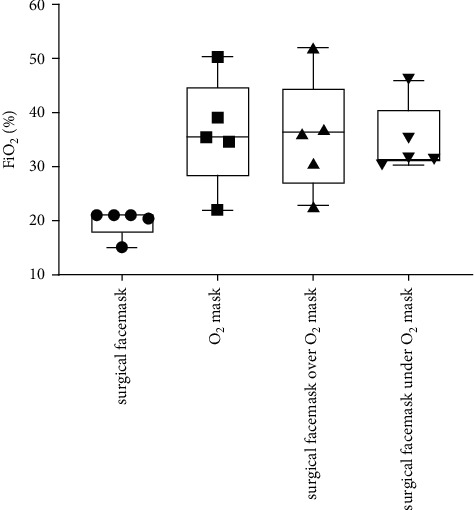
Box and Whisker plots of sampled FiO_2_ at the lips with a surgical facemask only, an O_2_ mask only, a surgical facemask over an O_2_ mask, and a surgical facemask under an O_2_ mask. The circles, squares, and triangles represent average values for each of the five subjects in the various mask configurations.

**Figure 2:**
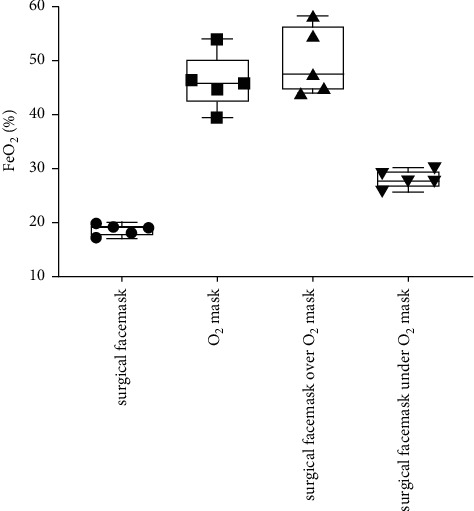
Box and Whisker plots of sampled FeO_2_ at the lips with a surgical facemask only, an O_2_ mask only, a surgical facemask over an O_2_ mask, and a surgical facemask under an O_2_ mask. The circles, squares, and triangles represent average values for each of the five subjects in the various mask configurations.

**Figure 3:**
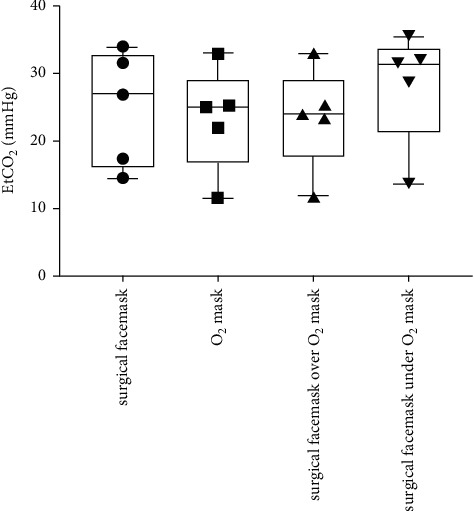
Box and Whisker plots of sampled EtCO_2_ at the lips with a surgical facemask only, an O_2_ mask only, a surgical facemask over an O_2_ mask, and a surgical facemask under an O_2_ mask. The circles, squares, and triangles represent average values for each of the five subjects in the various mask configurations.

**Figure 4:**
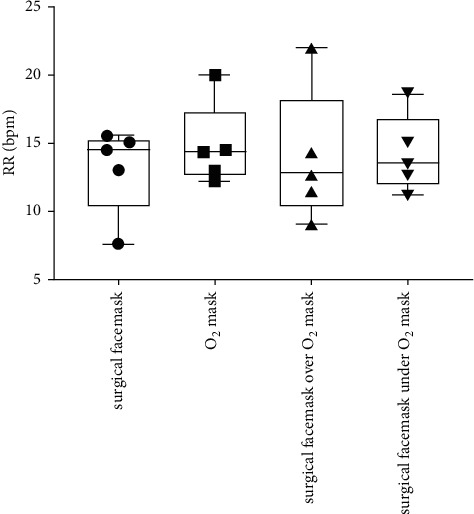
Box and Whisker plots of respiratory rate (RR) measured as breaths per minute computed using capnography with a surgical facemask only, an O_2_ mask only, a surgical facemask over an O_2_ mask, and a surgical facemask under an O_2_ mask. The circles, squares, and triangles represent average values for each of the five subjects in the various mask configurations.

Table 1 and Figures 1–4 show the measured FiO_2_, FeO_2_, expired EtCO_2_, and respiratory rate for the various surgical facemask and O_2_ mask configurations: a surgical facemask only, an O_2_ mask only, a surgical facemask under an O_2_ mask, and a surgical facemask over an O_2_ mask.

### 3.1. FiO_2_ Is Unchanged Regardless of Surgical Facemask Configuration with the O_2_ Mask

Figure 1 shows there was no significant difference in the sampled FiO_2_ at the lips when the surgical facemask was under the O_2_ mask compared to when the surgical facemask was over the O_2_ mask (34.8 ± 6.5 vs. 35.7 ± 10.7, *p*=0.885), as well as with the O_2_ mask alone (34.8 ± 6.5 vs. 36.3 ± 10.2, *p*=0.795).

As expected, the sampled FiO_2_ at the lips was significantly lower with the surgical facemask on room air compared to when the surgical facemask was over (19.7 ± 2.6 vs. 35.7 ± 10.7, *p*=0.035) or under the O_2_ mask (19.7 ± 2.6 vs. 34.8 ± 6.5, *p* < 0.01), as well as the O_2_ mask alone (19.7 ± 2.6 vs. 36.3 ± 19.2, *p*=0.040).

### 3.2. FeO_2_ Is Reduced When the Surgical Facemask is under Compared to over the O_2_ Mask

Figure 2 shows that the sampled FeO_2_ at the lips was significantly lower when the surgical facemask was under the O_2_ mask compared to when the surgical facemask was over the O_2_ mask (27.9 ± 1.68 vs. 49.9 ± 6.27, *p*=0.001). The FeO_2_ when the subject had only a surgical facemask was significantly higher compared to when the surgical facemask was under the O_2_ mask (18.6 ± 1.1 vs 27.9 ± 1.68, *p* < 0.001). There was no significant difference in sampled FeO_2_ when the surgical facemask was over the O_2_ mask compared to the O_2_ mask alone (49.9 ± 6.3 vs. 46.1 ± 5.2, *p*=0.864).

### 3.3. Expiratory EtCO_2_ Increased with the Surgical Facemask under Compared to over the O_2_ Mask

Figure 3 shows that the sampled expiratory EtCO_2_ was significantly higher when the surgical facemask was under the O_2_ mask compared to when the surgical facemask was over the O_2_ mask (28.3 ± 8.5 vs. 23.5 ± 7.6, *p*=0.026) and the O_2_ mask alone (28.3 ± 8.5 vs. 23.3 ± 7.8, *p*=0.010). There was no significant different in expiratory EtCO_2_ with the surgical facemask under the O_2_ mask compared to the surgical facemask alone (28.3 ± 8.5 vs. 24.9 ± 8.6, *p*=0.36).

### 3.4. RR Is Unchanged Regardless of Surgical Facemask and O_2_ Mask Configuration

The RR was not significantly changed when the surgical facemask was under compared to over the O_2_ mask (14.2 ± 2.8 vs. 13.9 ± 4.9, *p*=0.88), as well as the O_2_ mask only (14.2 ± 2.8 vs. 14.8 ± 3.1, *p*=0.70).

## 4. Discussion

The ability to provide supplemental oxygen and ensure adequate ventilation is essential for patients recovering from anesthesia and surgery. The COVID-19 pandemic has challenged providers to meet patients' needs for adequate gas exchange while minimizing the risks of infectious aerosolization. While guidelines recommend that patients wear a facemask in addition to their oxygen mask or nasal cannula, there is limited literature on the most effective way of achieving oxygenation and ventilation goals. Our study examines the various effects on oxygenation and ventilation of patients with the surgical facemask under or over the O_2_ mask and shows for the first time the consequences of reduced FeO_2_ and increased expired EtCO_2_ with the surgical facemask under compared to over an O_2_ mask in healthy volunteers. These effects may have significant consequences, particularly in patients with marginal respiratory reserve and the ability to tolerate apneic episodes without significant consequences during the postoperative period such as during transport.

A small number of prior investigations [3–7] have examined the effects of the face mask or nasal cannulae in combination with a surgical mask on oxygen delivery. These prior studies have been limited by small numbers of subjects, lack of repeated measurements, and focus on specific measures of gas exchange. An initial investigation by Binks et al. measured the FiO_2_ at the lips in a healthy volunteer breathing 6 L/min oxygen via an (Hudson) O_2_ mask placed over the top of a surgical mask and breathing 6 L/min oxygen via an O_2_ mask placed underneath a surgical mask. The FiO_2_ measured for the 2 mask configurations were 0.50 and 0.54, respectively, and the authors concluded that the O_2_ mask can be worn over a surgical facemask without compromising the FiO_2_. It is important to note that this study did not evaluate differences in the effects on FeO_2_ between the 2 mask configurations which we found were reduced with the mask under rather than over the O_2_ mask despite no significant change in measured FiO_2_. The reduced FeO_2_ with the surgical facemask under compared to over the O_2_ mask is likely the result of the relatively reduced effective flow rate of oxygen and increased entrainment of air resulting in reduced effective O_2_ delivery to the lungs. Of note, it is the FeO_2_ rather than the FiO_2_ that reflects the oxygen stores within the lung and increases the safety factor if apnea occurs [4].

Matsui et al. compared the impact on oxygenation and ventilation in a single subject wearing an oxygen mask above a surgical mask and wearing a nasal cannula below a surgical mask [5]. The authors found that the partial pressure of oxygen in arterial blood (PaO_2_) decreased when changing from the nasal cannula at 4 L/min to the oxygen mask 4 L/min (from 154 mmHg to 108 mm Hg), recovering once again when returning to the nasal cannula. In contrast, the partial pressure of carbon dioxide in arterial blood (PaCO_2_) and respiratory rate remained almost unchanged. The authors concluded that oxygen delivered via a nasal cannula worn under a surgical mask might prevent the spread of infection while simultaneously allowing maintenance of a high PaO_2_ in patients. Notably, their investigation was limited by the single subject design and lack of repeated measurements.

Montiel et al. examined the impact of placing surgical facemasks on patients that were receiving a high-flow nasal cannula (HFNC) for hypoxemic respiratory failure due to COVID-19 [6]. Among the 21 patients studied, the investigators found the PaO_2_ increased from 59 (±6) to 79 mmHg (±16) (*p* < 0.001) when a surgical facemask was placed over HFNC. The investigators postulated that the improvement in oxygenation could be explained not only by an increased oxygen concentration under the mask but also by a decrease of room air entrainment that is known to dilute the gas mixture with less inspired O_2_ concentration. Notably, the investigation did not evaluate the delivery of HFNC delivered in front of a surgical mask.

Hamada et al. measured FiO_2_ around the lips through the mouth under normal breathing conditions in five healthy volunteers in three situations: an O_2_ mask alone, a surgical facemask over an O_2_ mask, or a surgical facemask under an O_2_ mask with O_2_ flow rates set at 5 L/min, 7 L/min, or 10 L/min [7]. The investigators found that regardless of the oxygen flow rate, the FiO_2_ was higher when wearing a surgical facemask over an oxygen mask compared to wearing a surgical mask under a surgical mask. In our study, we found that FiO_2_ was not significantly changed when the surgical facemask was placed under the O_2_ mask compared to over the O_2_ mask; however, we used a simple O_2_ mask, whereas it appears Hamada et al. used a nonrebreather O_2_ mask as they were able to achieve nearly an FiO_2_ of 80% when the surgical facemask was over their O_2_ mask in their study. To our knowledge, our study is the first to examine the impact on oxygenation and ventilation of asurgical facemask placed over compared with under the O_2_ mask configurations among multiple subjects with repeated measurements. Importantly, our study demonstrated that ventilation was less effective with the surgical mask under the oxygen mask as indicated by the increase in CO_2_. It might be postulated that the increase in CO_2_ would be more pronounced in patients with less effective ventilation (e.g., lung disease or residual anesthetic effects) than what was seen in the healthy volunteers in our study. Our study could also have implications for nonsurgical patients in other areas of the hospital.

Our study was limited by the small number of subjects included, use of healthy subjects only, and fixed oxygen flow rate. Our results might be more pronounced in patients with underlying lung disease or those with respiratory impairment due to the residual effects of anesthetics, analgesics, and paralytics. A more prolonged duration of time with a given mask configuration might also have differing effects.

## 5. Conclusions

Effective oxygen delivery and ventilation were impaired (reduced FeO_2_ and increased EtCO_2_) when a surgical facemask was placed under compared to over an O_2_ mask. These findings may have important implications for ensuring adequate gas exchange for patients wearing both oxygen and surgical masks to minimize viral aerosolization. Further studies which include a larger number of participants and those with limited respiratory drive or reserve are needed to more fully evaluate the clinical significance of surgical and oxygen mask configurations on oxygen delivery and ventilation.

## Figures and Tables

**Table 1:** Table of measured FiO_2_, FeO_2_, EtCO_2_, and respiratory rate with a surgical facemask only, an O_2_ mask only, a surgical facemask over an O_2_ mask, and a surgical facemask under an O_2_ mask. Values are reported at mean ± standard deviation.

	FiO_2_ (%)	FeO_2_ (%)	EtCO_2_ (mmHg)	RR (bpm)
Surgical facemask	19.7±2.6	18.6±1.2	24.9±8.6	13.1±3.2
O_2_ mask	36.3±10.2	46.01±5.2	23.3±7.8	14.8±3.1
Surgical facemask over an O_2_ mask	35.7±10.7	49.9±6.3	23.5±7.6	13.9±4.9
Surgical facemask under an O_2_ mask	34.8±6.5	27.9±1.7	28.3±8.5	14.2± 2.8

## Data Availability

All data points are plotted in the figures and are available on request.

## Additional Points

*Take Home Message.* The COVID-19 pandemic has challenged providers to meet patients' needs for adequate gas exchange while minimizing the risks of infectious aerosolization. While guidelines recommend that patients wear a facemask in addition to their oxygen mask or nasal cannula, there is limited literature on the most effective way of achieving oxygenation and ventilation goals. Our study examines the various effects on oxygenation and ventilation of patients with the surgical facemask under or over the O_2_ mask and shows for the first time the consequences of reduced FeO_2_ and increased expired EtCO_2_ with the surgical facemask under compared to over an O_2_ mask in health volunteers. These effects may have significant consequences particularly in patients with reducedrespiratory drive or reserve and the ability to tolerate apneic episodes without significant consequences during the postoperative period such as during transport.
